# Contribution of Brain Processes to Tissue Loss After Spinal Cord Injury: Does a Pain-Induced Rise in Blood Pressure Fuel Hemorrhage?

**DOI:** 10.3389/fnsys.2021.733056

**Published:** 2021-12-15

**Authors:** Gizelle N. K. Fauss, Misty M. Strain, Yung-Jen Huang, Joshua A. Reynolds, Jacob A. Davis, Melissa K. Henwood, Christopher R. West, James W. Grau

**Affiliations:** ^1^Department of Psychological and Brain Sciences, Texas A&M University, College Station, TX, United States; ^2^Department of Cellular and Integrative Physiology, University of Texas Health Science San Antonio, San Antonio, TX, United States; ^3^Sundia MediTech Co., Ltd., Shanghai, China; ^4^Centre for Chronic Disease Prevention and Management, Faculty of Medicine, University of British Columbia, Kelowna, BC, Canada

**Keywords:** spinal cord injury, pain, polytrauma, hemorrhage, blood pressure, cardiovascular function, spinal transection, norepinepherine

## Abstract

Pain (nociceptive) input soon after spinal cord injury (SCI) expands the area of tissue loss (secondary injury) and impairs long-term recovery. Evidence suggests that nociceptive stimulation has this effect because it promotes acute hemorrhage. Disrupting communication with the brain blocks this effect. The current study examined whether rostral systems exacerbate tissue loss because pain input drives an increase in systolic blood pressure (BP) and flow that fuels blood infiltration. Rats received a moderate contusion injury to the lower thoracic (T12) spinal cord. Communication with rostral processes was disrupted by cutting the spinal cord 18 h later at T2. Noxious electrical stimulation (shock) applied to the tail (Experiment 1), or application of the irritant capsaicin to one hind paw (Experiment 2), increased hemorrhage at the site of injury. Shock, but not capsaicin, increased systolic BP and tail blood flow in sham-operated rats. Cutting communication with the brain blocked the shock-induced increase in systolic BP and tail blood flow. Experiment 3 examined the effect of artificially driving a rise in BP with norepinephrine (NE) in animals that received shock. Spinal transection attenuated hemorrhage in vehicle-treated rats. Treatment with NE drove a robust increase in BP and tail blood flow but did not increase the extent of hemorrhage. The results suggest pain input after SCI can engage rostral processes that fuel hemorrhage and drive sustained cardiovascular output. An increase in BP was not, however, necessary or sufficient to drive hemorrhage, implicating other brain-dependent processes.

## Introduction

After initial insult to the spinal cord (primary injury), the area of cell death expands, potentially doubling the area of injury (secondary injury). The extent of primary injury varies with the severity of impact to the spinal cord and is irreversible. Secondary injury however, depends on the neurobiological processes that unfold over hours to days following the primary injury (McVeigh, 1923; Ducker et al., 1971; Beattie et al., 2002; Hausmann, 2003). Microglial activation, inflammation, vascular destruction, and necrotic cell death have been shown to contribute to the deleterious consequences of secondary injury (Mautes et al., 2000; Alizadeh et al., 2019). Expansion of secondary injury has been linked to symptoms of allodynia, hyperalgesia, and poor functional recovery (Hook et al., 2017; Turtle et al., 2017). Indeed, current research suggests that these dynamic processes can not only be alleviated but prevented (Simard et al., 2010; Lee et al., 2014, 2015, 2018). By targeting the mechanisms that underlie secondary injury, we can potentially suppress the expansion of cell death and improve functional outcomes.

Through the use of a clinically relevant animal (rat) model, we have explored the effects of pain (nociceptive) input on secondary injury using electrical stimulation applied to the tail at an intensity that engages C-fibers (Crown et al., 2002; Ferguson et al., 2006; Baumbauer et al., 2008) or applying the irritant capsaicin to the hind paw (Turtle et al., 2018). We have shown that nociceptive input soon after a contusion injury (24–96 h) expands the area of secondary injury and impairs long-term recovery (Grau et al., 2004; Turtle et al., 2017, 2018). Recent work has linked the latter effects to nociceptive-induced hemorrhage at the injury site (Turtle et al., 2019). Following noxious stimulation, histopathological analyses revealed evidence of capillary fragmentation, the upregulation of the Sur1-Trpm4 channel complex known to be involved in the development of hemorrhage and enlarged areas of tissue loss (Turtle et al., 2019). The acute failure of the microvasculature suggests the involvement of the auto-destructive process known as progressive hemorrhagic necrosis (PHN) that results in the expansion of the hemorrhagic lesion (Simard et al., 2013). This effect has been linked to the breakdown of the BSCB, which allows blood to infiltrate and cause further damage to the neural tissue (Lee et al., 2014, 2015).

Other studies have related tissue loss after injury to a rise in blood pressure (BP; Guha et al., 1989; Nielson et al., 2015). Noxious electrical stimulation has been shown to induce an elevation in BP (Snow et al., 1978; Lindan et al., 1980; Karlsson, 1999; Canon et al., 2015) which could increase blood-brain barrier permeability (Heistad and Marcus, 1979; Ito et al., 1980; Hardebo and Beley, 1984). The hemodynamic response to noxious stimulation could be mediated by direct projections from the mid-thoracic (T1–T6) spinal cord through sympathetic fibers (Krassioukov et al., 2003; Rabchevsky, 2006). Indeed, when communication with the brain is cut by means of rostral (e.g., T2) transection, noxious stimulation can lead to an unregulated spike in BP and heart rate (HR), a process that is symptomatic of autonomic dysreflexia (AD; Marsh and Weaver, 2004; Eldahan and Rabchevsky, 2018). Alternatively, brain-dependent processes could drive a destructive rise in BP. Spinal cord injury also leads to dysregulation of the hypothalamic-pituitary-adrenal (HPA) axis, leading to increased release of stress hormones and other systemic effects that could affect tissue survival at the site of SCI (Lucin et al., 2007, 2009).

In a prior article, we examined whether systems rostral to T2 impact pain-induced hemorrhage after SCI by surgically cutting (transecting) communication with the brain at T2 18 h after animals received a lower thoracic (T12) contusion injury (Reynolds et al., 2019). Based on prior work (Crown and Grau, 2005), we hypothesized that brain systems exert a homeostatic effect that would counter pain-induced overexcitation and cell death at the site of injury. Contrary to our expectations, a T2 transection blocked shock-induced activation of pro-inflammatory cytokines[tumor necrosis factor, interleukin 1ß (IL-1ß), IL-18] and signals related to cell death (caspase 1; Reynolds et al., 2019). A T2 transection also blocked shock-induced hemorrhage. The present paper extends this work by showing that a T2 transection blocks capsaicin-induced hemorrhage (Experiment 2).

The fact a rostral spinal cord transection blocks nociception induced hemorrhage implicates afferent sensory fibers, that engages a brain-dependent process that fuels the break-down of the blood spinal cord barrier and the subsequent infiltration of blood at the site of injury. We hypothesized that this destructive effect may be linked to a systemic rise in systolic blood pressure and/or flow, engaged by rostral processes in response to pain and/or stress. If this is true, a spinal transection should block both increased cardiovascular output and hemorrhage (Experiments 1 and 2). Conversely, artificially driving a rise in BP, by pretreating animals with the adrenergic agonist norepinephrine (NE), should substitute for the brain-dependent effect, driving hemorrhage in transected rats exposed to shock (Experiment 3). Alternatively, nociceptive input may impact cardiovascular function through sympathetic fibers caudal to T2. In this case, noxious stimulation would be expected to drive a rise in blood pressure in transected rats and imply that the brain produces hemorrhage by engaging other processes.

## Materials and Methods

### Subjects

Adult male Sprague-Dawley rats (*N* = 108), purchased from Envigo (Houston, Texas) were used. They weighed 375 g ± 3.09 (mean ± SE) and were acclimated to their holding environment for at least 7 days prior to experimentation. Prior to surgery, rats were dual housed, with food and water *ad libitum* and maintained on a 12-h light/dark cycle with all behavioral testing performed during the light cycle. All experiments were carried out in accordance with NIH standard for the care and use of laboratory animals (NIH publications No. 80-23) and were approved by the University Laboratory Animal Care Committee at Texas A&M University. Every effort was made to limit the number of animals and minimize unnecessary suffering.

### Spinal Contusion

Rats were anesthetized with a 5% isoflurane and maintained at 2–3% isoflurane during surgery. After they were shaved and the surgical site cleaned with iodine and alcohol, a single longitudinal incision was made, extending approximately 3 cm over to the injury site. Two longitudinal incisions were made on either side of the vertebral column cut to the depth of the rib cage. One cm lateral incisions were made immediately below the T10 vertebra and above the T13 vertebra. After clearing the tissue, a laminectomy was performed at the T10-T11 vertebral level. The New York University (NYU) Multicenter Animal Spinal Cord Injury Study (MASCIS) device was used to perform the contusion injury (Gruner, 1992). Clamps were used to secure the spinal cord, and the 10-g impactor was centered on the lesion site. The drop height was set at 12.5 mm. After impact, the wound was closed with Michel clips. To prevent urinary tract infection and compensate for fluid loss, animals received 100,000 units/kg of penicillin and 3 ml of saline intraperitoneal (i.p.) after surgery.

Animals were given 18 h to recover in a temperature-controlled room (25°C). Food and water were available *ad libitum*. The rats’ bladders were expressed twice daily and immediately after all BP time points. After experimentation was complete, all animals were euthanized with a lethal dose of pentobarbital [100 mg/kg; i.p.].

### Spinal Transection

The animals underwent a spinal transection 18 h after the contusion injury. Anesthesia was induced with 5% isoflurane gas and maintained at 2–3% isoflurane throughout the procedure. Their heads were then secured in a stereotaxic apparatus. The skin over the upper thoracic region was shaved and disinfected with iodine and alcohol. A longitudinal incision was made over the second thoracic vertebrae (T2) and the tissue just rostral to T2 was cleared away. At this point, the sham rats had their wound closed with Michel clips. The remaining rats had their spinal cord transected with a cautery device and the wound was closed using Michel clips. All animals then received 3 ml of saline (i.p.). The transections were visually confirmed post-mortem at the time of sacrifice.

### Uncontrollable Electrical Stimulation

Rats were loosely restrained in an opaque Plexiglass tube and placed in an acoustic isolation chamber. An electrode, fashioned from a modified fuse clip, was coated with electrode paste and taped to the tail. Animals received six minutes of electrical stimulation applied through the electrode to the tail (180 stimuli; 100 ms; 0.2–3.8 s ISI) at an intensity known to engage C-fibers (1.5 mA; Thompson et al., 1990; Hathway et al., 2009). Rats in the unshock group received the same treatment without the electrical stimulation.

### Peripheral Capsaicin Injection

Rats were loosely restrained in an opaque plexiglass tube and 3 percent capsaicin dissolved in vehicle solution [Tween-20 (5%), EtOH (5%), and saline (90%)] was injected (50 μl) intradermally into the dorsal surface of the hind paw with a 27-gauge needle. Controls were injected with an equal volume of vehicle solution. Animals were randomly injected on the left or right paw. Animals were left in the plexiglass tubes for 6 min to equate the treatment time across experiments.

### Non-invasive Blood Pressure Measurement

Testing was performed in a warm room (27°C) with dim lighting. Rats were placed in a clear acrylic tube with a black adjustable nose cone atop a warming platform (Kent Scientific). The rats were acclimated to the BP apparatus for 5 min. Then, an occlusion cuff and Volume Pressure Recording (VPR) cuff were secured at the base of the tail. After five additional minutes of acclimation to the apparatus and cuffs, the subjects underwent 15 cycles of BP measurement (Kent Scientific). Blood pressure was obtained using the CODA High Throughput Noninvasive Blood Pressure system and data acquisition software. The program was set to automatically inflate and deflate the cuffs for 15 s with 5 s between each cycle. The maximum occlusion pressure was set at 250 mmHg with a deflation time of 20 s and a minimum volume of 15 μl. Tail temperature was monitored with an infrared thermometer aimed at the base of the tail. Care was taken to maintain the rats at a constant temperature (approximately 33°C) during testing with a warming blanket placed over the tail, when necessary, to increase temperature. To cool the rats, a dampened paper towel was placed over the tube. Blood pressure was assessed in the minutes before treatment and 0, 1, 2, and 3 h after. This non-invasive BP method has been validated for accuracy of measurement in rodents (Feng et al., 2008).

Six measures of cardiovascular function were obtained: systolic BP, diastolic BP, mean arterial BP, heart rate, tail blood flow, and tail blood volume. Preliminary analyses of the baseline values, and the change observed after treatment, showed that the three measures of BP were highly correlated (all *r*’s > 0.905, *p* < 0.0001). Due to this redundancy, only one measure (systolic BP) is presented. Likewise, tail blood flow and volume were highly correlated (*r*’s > 0.954, *p* < 0.0001). For this reason, and because tail blood flow has proven to be more reliably related to our experimental effects, we present blood flow. Finally, because a pain-induced rise in heart rate was not strongly correlated with the changes observed in systolic BP and blood flow (all *r*’s < 0.404), it too is presented.

### Norepinephrine Injection

After shock treatment, animals were transferred to BP tubes and given a 2 ml subcutaneous injection of norepinephrine (0.1 mg/kg) or vehicle (saline) to the trunk. The first assessment of BP was conducted immediately after administration of drug. The dosage used was based on pilot work demonstrating it produces an increase in systolic BP comparable to that induced by noxious electrical stimulation (Johnston et al., 2021).

### Behavioral Testing

Baseline locomotor performance was scored 18 h after SCI, and before the transection surgery, using the Basso, Beattie, and Bresnahan (BBB) scale (Basso et al., 1995). In all cases, there were no group differences prior to spinal transection (all *F*s <2.73, *p* > 0.05). BBB was not assessed after spinal transection due to the complete nature of the injury.

### Tissue Collection and Protein Extraction

Rats were sacrificed with a lethal injection of pentobarbital (100 mg/kg; i.p.). One cm of spinal tissue centered over the injury site was collected. The collected tissue was then flash frozen in liquid nitrogen and stored in −80°C. The protein was isolated from the collected spinal tissue using Trizol RNA extraction followed by a protein extraction procedure using the Qiagen kit.

### Spectrophotometry

Approximately 1.5 μl of protein extract was placed on the spectrophotometer (NanoDrop, Thermo Scientific), and aa full spectral analysis was done [from 200 to 800 nanometers (nm)]. Absorbance values at 420 nm, run in triplicate, were used as a measure for hemoglobin content (Turtle et al., 2019).

Hemoglobin content at the site of injury was also assessed with Drabkin’s assay, which is based on cyanomethemoglobin colorimetry (Sadie, 1920; van kampen and Zijlstra, 1961; Choudhri et al., 1997). Twenty microliter of sample protein was processed in 80 μl of a Drabkin’s-Tween20 solution (2% Tween20). The samples were then sonicated for 30 s on ice and incubated for 30 min. After centrifugation (10,000 *g* at 15°C for 25 min), 1.5 μl of the sample was placed on the spectrophotometer and measured at 540 nm for blood content.

### Western Blot

The concentration of the extracted protein was measured using the Bradford assay. The samples were diluted to a final concentration of 3 μg/μl in 4× Lamelli buffer. Western blotting was performed using a 26-well 12% Tris-HCL Criterion precast gels (BioRad, Hercules, CA) according to manufacturer’s instructions. The diluted samples were heated to 95°C for 10 min and centrifuged for a quick spin cycle (3–5 s). Then, equal amounts of the protein (30 μg) were loaded into each well. After the addition of SDS-PAGE running buffer, electrophoresis was performed at 180 V for approximately one hour. Proteins were then transferred onto a polyvinylidene difluoride (PVDF; Millipore, Bedford, MA) membrane for one hour in an ice bucket at 100 V in cold transfer buffer. The membrane was then blocked in 5% blotting-grade milk (BioRad, Hercules, CA) for one hour prior to overnight incubation in primary antibodies hemoglobin α [1:1,000; Abcam (Cambridge, MA) ab92492; RRID: AB10561594], Lamin B1 (1:1,000; Abcam ab16048; RRID: AB443298), and Beta Tubulin [1:5,000; Upstate (Lake Placid, NY) RRID: AB309885 at 4°C. After three washes in Tris-buffered saline and Tween-20 (TBST) at 10 min each, the blots were incubated for one hour in HRP-conjugated goat anti-rabbit secondary antibodies [1:5,000; Thermo Scientific (Rockford, IL); RRID: AB228341 or HRP-conjugated goat anti-mouse [1:5,000; Pierce Biotech (Rockford, IL); RRID: AB258492] at room temperature. Finally, the blots were washed for another 3 × 10 min series in TBST and developed using electrochemiluminescence (ECL; Pierce, Rockford, IL). The blots were imaged with Fluorchem HD2 (ProteinSimple, Santa Clara, CA) and were analyzed by calculating the ratios of the integrated densitometry of each protein of interest to the loading control (lamin B1 or beta tubulin), then normalizing this ratio to a control group (run on the same blot) that did not receive nociceptive treatment (in the first two experiments) or drug (in experiment 3).

### Experimental Designs

#### Experiment 1

**Figure 1:**
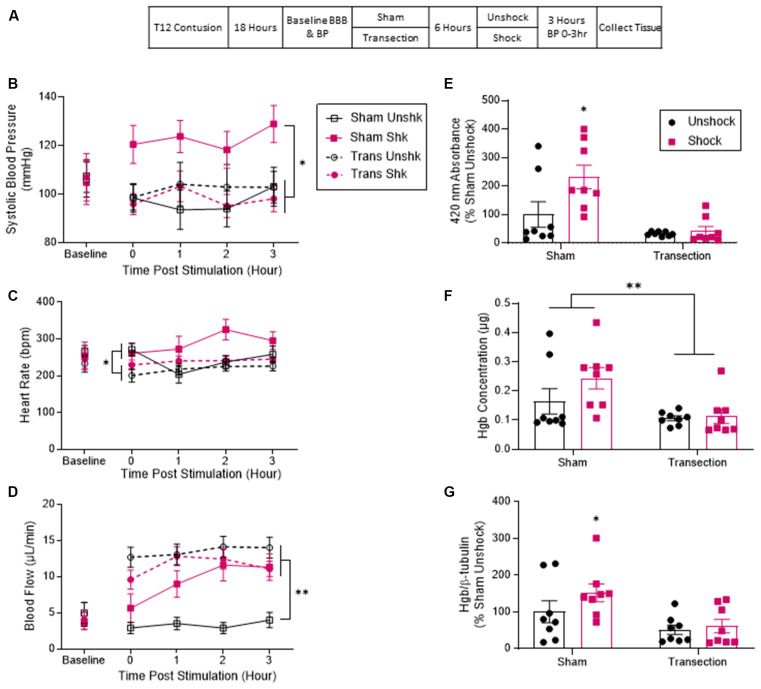
Noxious electrical stimulation increased BP and hemorrhage after a lower thoracic contusion injury and this effect was blocked by a spinal transection. **(A)** Experimental design and timeline for experiment 1. **(B)** Sham-operated rats that received electrical stimulation (Shk) exhibited higher systolic blood pressure over the next 3 h (T0–T3). Transection surgery blocked this effect. **(C)** Rats treated with electrical stimulation exhibited higher heart rate over the 3 h. **(D)** Both transected groups and the sham-operated rats that received shock displayed a significant increase in tail blood flow. Sham-operated unshocked animals remained unchanged. **(E)** Quantification of peak absorbance at 420 nm (the wavelength associated with hemoglobin). Sham shocked rats showed a higher peak absorbance than unshocked rats. Transection surgery blocked this effect. **(F)** Quantification of hemoglobin content based on formation of cyanomethemoglobin (Drabkin’s assay). Tissue from sham shocked rats contained a higher concentration of hemoglobin relative to animals that had undergone a spinal transection. **(G)** Immunoblot quantification for hemoglobin showed that tissue samples from sham-operated rats that received shock had higher levels of hemoglobin relative to both the sham-operated unshocked group and both groups that received a transection. Asterisks indicate statistical significance (*p < 0.05, **p < 0.01, *n* = 8). An asterisk placed over a group indicates that the group differs from all the others. Error bars represent the standard error of the mean (SEM).

Thirty-two rats received a moderate T12 contusion injury and eighteen hours later, half of the subjects were randomly assigned to undergo a T2 transection surgery. Twenty-four hours after the original contusion injury, half of the animals in each group received six minutes of intermittent shock to the tail or an equal period of restraint (Figure 1A). Cardiovascular function was assessed immediately before spinal transection and at hourly intervals for 3 h after shock treatment. Finally, rats were sacrificed for tissue collection. The full design involved a full 2 (Sham vs. Transection) × 2 (Shock vs. Unshock) factorial (*n* = 8).

#### Experiment 2

**Figure 2:**
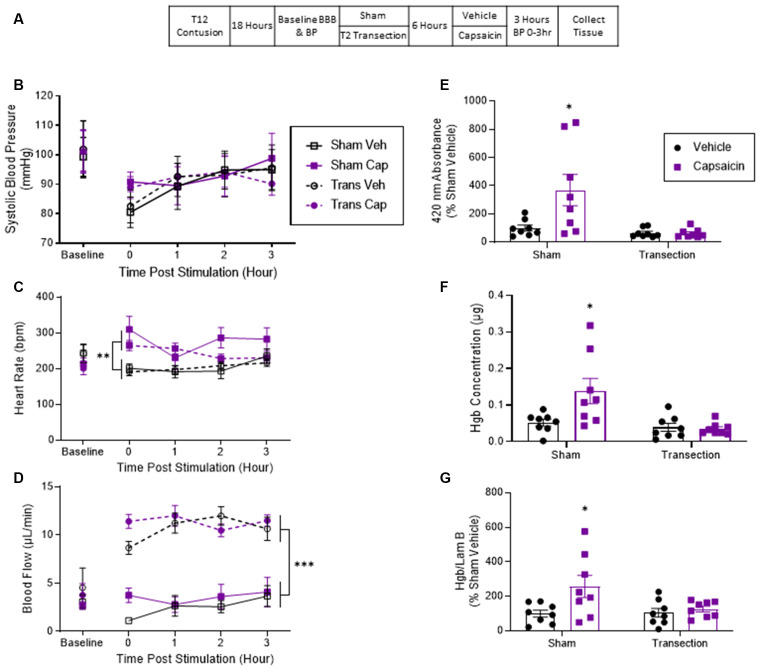
Application of the irritant capsaicin to a hind paw increased hemorrhage after a lower thoracic contusion injury and this effect was blocked by a spinal transection. **(A)** Experimental design and timeline for experiment 2. **(B)** Application of capsaicin (Cap) to one hind paw did not induce a change in systolic blood pressure. **(C)** Capsaicin-treated rats exhibited a higher heart rate throughout the 3 h. **(D)** Only transected rats exhibited a significant rise in tail blood flow after capsaicin treatment. **(E)** Sham-operated rats that were treated with capsaicin exhibited greater absorbance at 420 nm for hemoglobin. Transection surgery blocked this effect. **(F)** Drabkin’s assay and western blot **(G)** showed similar results. Asterisks indicate statistical significance (*p < 0.05, **p < 0.01, ***p < 0.001, *n* = 8). An asterisk placed over a group indicates that the group differs from all the others. Error bars represent the standard error of the mean (SEM).

Thirty-two rats received a contusion injury and 18 h later half the subjects (randomly assigned) had the spinal cord transected at T2. The remaining contused rats underwent a sham surgery. Six hours later, half of the animals in each condition were treated with capsaicin while the remaining rats received vehicle. BP and tail blood flow were assessed as described above. Three hours after capsaicin treatment, the animals were sacrificed and tissue was collected (Figure 2A). The experiment involved a 2 (Sham vs. Transection) × 2 (Capsaicin vs. Vehicle) factorial design (*n* = 8).

#### Experiment 3

**Figure 3:**
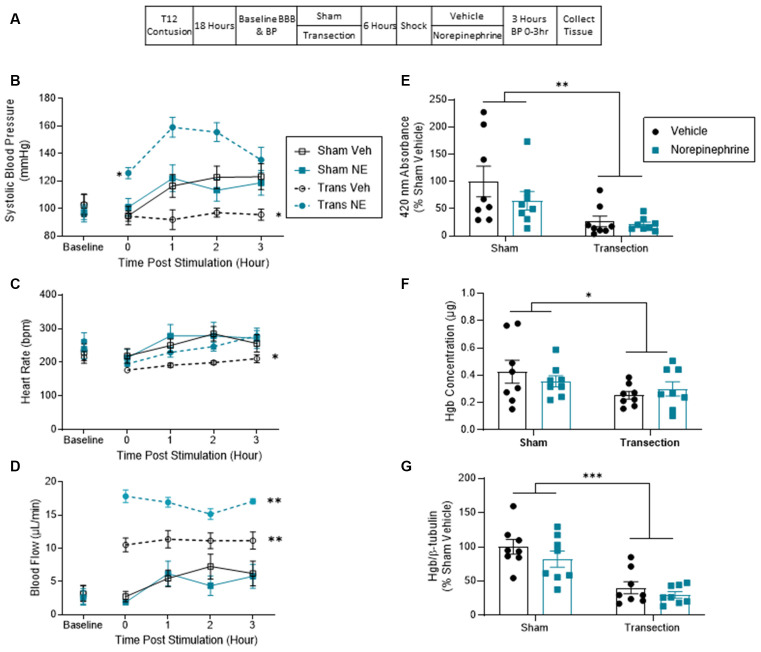
Norepinephrine increased blood pressure in transected but not sham-operated rats. This significant rise did not induce hemorrhage at the injury site. **(A)** Experimental design and timeline for experiment 3. **(B)** Sham-operated shocked rats exhibited a rise in systolic blood pressure relative to vehicle-treated shocked animals that were transected. Transected rats that were given NE exhibited the greatest increase in BP. **(C)** Sham-operated rats exhibited greater heart rate throughout testing. Transected rats that received vehicle exhibited the lowest heart rate throughout the 3 h. **(D)** Spinally transected animals exhibited higher levels of tail blood flow, relative to sham-operated rats. NE increased tail blood flow in transected, but not sham-operated, rats. **(E)** Spectrophotometry results at 420 nm for hemoglobin revealed that sham-operated shocked rats exhibited greater absorbance, relative to the transected animals. NE had no effect. **(F)** Drabkin’s assay and western blot **(G)** showed a similar pattern. Only sham-operated rats showed an increase in hemoglobin content and expression at the injury site. Asterisks indicate statistical significance (*p < 0.05, **p < 0.01, ***p < 0.001, *n* = 8). An asterisk placed over a group indicates that the group differs from all the others. Error bars represent the standard error of the mean (SEM).

Thirty-two rats received a contusion injury and were randomly assigned to receive either a rostral transection surgery or sham surgery 18 h later. Six hours later, BP was assessed and all the rats in each condition received shock. Next, half of the rats in each condition were injected with NE. The remaining animals were given its vehicle. Cardiovascular function and hemorrhage were assessed as described above (Figure 3A). The experiment involved a 2 (Sham vs. Transection) × 2 (NE vs. Vehicle) factorial design (*n* = 8).

### Statistics

All data were analyzed using analysis of variance (ANOVA) or analysis of covariance (ANCOVA). Differences between group means were assessed using Duncan’s New Multiple Range *post hoc* tests when necessary. To control for variability in labeling across blots, this factor was entered in our analyses for western blotting.

## Results

### Experiment 1: Spinal Transection Blocked the Shock-Induced Increase in Systolic Blood Pressure and Hemorrhage

Past work has shown that a rise in BP can increase tissue loss and impair long-term recovery after SCI (Guha et al., 1989; Nielson et al., 2015). Noxious stimulation leads to a rise in BP that could fuel hemorrhage (Snow et al., 1978; Canon et al., 2015). We hypothesized that cutting communication with the brain blocks the nociception-induced rise in systolic BP, protecting against the consequences of prolonged elevated BP. Alternatively, work on AD suggests that nociceptive input can induce a rise in BP, through the disruption of descending modulating pathways and the resulting unregulated control of sympathetic reflexes. To study this phenomenon, researchers typically cut communication with the brain by means of a high thoracic (T1–T6) transection, an experimental treatment that can enhance the effect of noxious stimulation on cardiovascular function (Laird et al., 2006; Rabchevsky et al., 2012; West et al., 2015, 2016). If this process contributes to the nociception-induced rise in BP in our paradigm, cutting the spinal cord should promote rather than block the effect.

Prior to transection surgery (Baseline), systolic BP ranged from 104.78 ± 9.68 to 107.52 ± 7.21 (mean ± SE) across groups. These differences were not statistically significant, all *F*s <1.0, *p* > 0.05. Analysis of systolic BP across the three hours showed that contused rats that were not transected (Sham) exhibited higher systolic BP after shock (Shk) treatment (Figure 1B). This phenomenon was blocked by a rostral transection. An analysis of covariance (ANCOVA), with baseline systolic BP serving as the covariate, confirmed that the effect of shock treatment depended upon whether animals had received a spinal transection, *F*_(1, 27)_ = 6.66, *p* = 0.0156 (*η*^2^ = 0.148). *Post hoc* comparisons of the group means showed that the sham-operated group that received shock (Sham Shk) differed from the other three (*p* < 0.05). No other group comparisons were significant (*p* > 0.05).

Baseline heart rate ranged from 233 ± 24.9 to 282 ± 20.4 (mean ± SE) across groups. These differences were not statistically significant, all *F*s <1.0, *p* > 0.05. The sham-operated shocked rats (Sham Shk) exhibited greater heart rate throughout the 3 h (Figure 1C). An ANCOVA with baseline heart rate serving as a covariate revealed a main effect of transection, *F*_(1, 27)_ = 6.336, *p* = 0.0181 (*η*^2^ > 0.177). *Post hoc* comparisons revealed a significant difference between the sham-operated shocked rats and the transected unshocked rats. There was also a main effect of time and an interaction between time and baseline heart rate, *F*s >2.79, *p* < 0.0458. No other comparisons were significant (*p* > 0.05).

Before spinal transection (Baseline), tail flow ranged from 3.64 ± 1.04–4.98 ± 4.58 (mean ± SE) and did not differ between groups, all *F*s <1.0, *p* > 0.05. Analysis of flow over the 3 h of testing showed that the sham-operated shocked rats (Sham Shk) and the transected rats (Trans Unshk and Trans Shk) exhibited an increase in tail blood flow after treatment (Figure 1D). An ANCOVA, with baseline flow serving as the covariate, revealed a between subjects main effect of transection surgery, and an interaction between shock treatment and transection, both *F*s >9.142, *p* < 0.0054 (*η*^2^ > 0.153). *Post hoc* comparisons of the group means revealed a significant difference between the sham-operated unshocked group (Sham Unshk) from the other three, and a difference between the sham-operated shocked group (Sham Shk) and transected unshocked group (Trans Unshk; *p* < 0.05). There was also a within subjects main effect of time, *F*_(3, 81)_ = 5.639, *p* = 0.0015, and a trend for significance between time and group (*p* = 0.0604). No other comparisons were significant (*p* > 0.05).

Blood content from tissue at the site of injury was first assessed by measuring absorbance at 420 nm (the absorbance peak for hemoglobin; Sadie, 1920; van kampen and Zijlstra, 1961; Choudhri et al., 1997; Turtle et al., 2019). Contused rats that were not transected exhibited greater absorbance relative to the sham unshocked group and both transected groups. Shock treatment had no effect on absorbance in transected rats. An ANOVA showed that the main effects of transection and shock treatment were statistically significant both *F*s > 5.060, *p* < 0.0325 (*η*^2^ > 0.094). The interaction between these variables approached significance, *F*_(1, 28)_ = 3.77, *p* = 0.0623. *Post hoc* comparisons confirmed that the non-transected (Sham) group that received shock differed from the other three groups (*p* < 0.05; Figure 1E).

A similar pattern was obtained with the Drabkin’s assay, with transected animals exhibiting lower hemoglobin content at the site of injury, *F*_(1, 28)_ = 9.046, *p* = 0.0055 (*η*^2^ > 0.067). *Post hoc* comparisons found that the two non-transected groups differed from the transected groups (*p* < 0.05; Figure 1F).

Alpha hemoglobin was also assessed using western blotting. Again, contused rats that were not transected (Sham) and received shock exhibited a higher concentration of hemoglobin relative to both the unshocked groups and transected rats that received shock (Figure 1G). An ANCOVA, with blot serving as a covariate, confirmed that shock and transection had a significant effect, both *F*s > 7.64, *p* < 0.01 (*η*^2^ > 0.082). *Post hoc* comparisons showed that the non-transected group given shock differed from the other three groups (*p* < 0.05). There were no other significant group comparisons (*p* > 0.05).

### Experiment 2: Spinal Transection Blocked Capsaicin-Induced Hemorrhage

Experiment 1 showed that nociceptive input caudal to a contusion injury produces an increase in BP and hemorrhage. Both effects were blocked by a rostral transection. To evaluate the generality of these findings, we tested another clinically relevant pain model, the irritant capsaicin. This irritant was chosen due to its common use in the pain literature (Simone et al., 1989; Lamotte et al., 1991; Hook et al., 2008; Huang et al., 2016) and its specific engagement of nociceptive fibers that express the TRPV1 receptor. In previous work, we have shown that capsaicin treatment induces enhanced mechanical reactivity (EMR) and engages cellular indices of nociceptive sensitization (Grau et al., 2012; Turtle et al., 2018). The present experiment examines whether capsaicin induces an increase in BP and hemorrhage, and whether these effects are blocked by spinal transection.

Prior to transection surgery (Baseline), systolic BP ranged from 99.27 ± 7.09 to 101.88 ± 10.31 (mean ± SE) across groups. These differences were not statistically significant, all *F*s <1.0, *p* > 0.05. Contused animals that were treated with capsaicin showed no change in BP independent of whether or not they received a spinal cord transection (Figure 2B). An ANCOVA confirmed that neither capsaicin nor spinal transection had a significant effect, all *F*s <1.0, *p* > 0.05.

Baseline heart rate ranged from 213 ± 17.8 to 243 ± 26.8 (mean ± SE) across groups. These differences were not statistically significant, all *F*s <2.3, *p* > 0.05. Rats that were treated with capsaicin displayed a greater heart rate throughout the 3 h (Figure 2C). An ANCOVA with baseline heart rate serving as the covariate revealed a main effect of capsaicin treatment, *F*_(1, 27)_ = 18.312, *p* = 0.0002 (*η*^2^ > 0.377). *Post hoc* comparisons of the group means showed that the two capsaicin-treated groups differed from those that received vehicle (*p* < 0.05). No other comparisons were significant (*p* > 0.05).

Before transection, tail blood flow ranged from 2.68 ± 0.417 to 4.54 ± 2.17 (mean ± SE) and did not differ between groups, all *F*s <1.0, *p* > 0.05. Analysis of tail blood flow over time showed that the transected rats had significantly higher flow than sham-operated rats (Figure 2D). An ANCOVA, with baseline flow serving as the covariate, revealed a main effect of transection surgery, *F*_(1, 27)_ = 120.922, *p* = 0.0001 (*η*^2^ > 0.796). *Post hoc* comparisons of the group means confirmed that the two transected groups differed from the non-transected (sham) animals (*p* < 0.05). No other comparisons were significant (*p* > 0.05).

Contused rats that had not undergone a spinal cord transection (Sham) exhibited greater absorbance at the wavelength associated with hemoglobin and this effect was blocked by spinal cord transection (Figure 2E). An ANOVA confirmed that the main effects of capsaicin and transection treatment, as well as their interaction, were statistically significant, all *F*s > 5.401, *p* < 0.0276; all *η*^2^ > 0.113). *Post hoc* comparisons confirmed that the sham group that received capsaicin differed from the other three groups (*p* > 0.05).

A similar pattern of results was obtained with the Drabkin’s assay. Again, capsaicin increased hemoglobin concentration at the site of injury in contused rats that were not transected (Sham) but not in contused and transected rats (Figure 2F). An ANOVA confirmed that the main effects of capsaicin and transection treatment, as well as their interaction, were statistically significant, all *F*s = 5.75, *p* < 0.05 (all *η*^2^ > 0.096). *Post hoc* comparisons showed that the sham-operated group that was treated with capsaicin differed from the other three (*p* < 0.05).

Western blotting confirmed that contused rats that received capsaicin had higher concentrations of hemoglobin at the site of injury relative to both the vehicle controls and transected rats that received capsaicin (Figure 2G). Because there was greater variability in behavioral performance after injury in this experiment, we analyzed the data using an analysis of covariance with baseline BBB score entered as a covariate. An ANCOVA revealed that there was a main effect of capsaicin, *F*_(1, 27)_ = 6.473, *p* = 0.017 (*η*^2^ > 0.160). *Post hoc* comparisons showed that the non-transected (Sham) group that received capsaicin differed from the other three groups (*p* < 0.05). No other group comparison was significant (*p* > 0.05).

### Experiment 3: Pharmacologically Increasing Blood Pressure in Transected Animals Does Not Lead to Hemorrhage

We found that noxious electrical stimulation and the irritant capsaicin increase hemorrhage at the site of injury, but only the former effect was accompanied by a rise in systolic BP. The fact that a rostral transection blocked both shock-induced hypertension and hemorrhage suggests the effects may be related. However, treatment with capsaicin had no discernable effect on systolic BP, but nevertheless increased hemorrhage at the site of injury. To further examine the relationship between BP and hemorrhage, we applied shock to rats that received a contusion injury and a rostral transection, and then pharmacologically induced a rise in BP with a systemic injection of norepinephrine (NE).

Prior to spinal transection (Baseline), systolic BP ranged from 95.95 ± 5.87 to 103.19 ± 7.83 (mean ± SE) across groups. These group differences were not statistically significant, all *F*s <1.0, *p* > 0.05. Shock induced an increase in BP in vehicle-treated rats that were not transected (Sham Veh) relative to vehicle-treated animals that were transected (Trans Veh) prior to shock treatment (Figure 3B). NE did not affect the shock-induced rise BP in the sham-operated group (Sham NE), but it did produce a robust effect in transected animals (Trans NE). An ANCOVA, with baseline BP serving as the covariate, confirmed that the main effect of NE treatment and its interaction with transection surgery were statistically significant, both *F*s > 14.828, *p* < 0.0007 (*η*^2^ > 0.253). *Post hoc* comparisons of the group means confirmed that the transected animals that received norepinephrine differed from the other three groups (*p* < 0.05). Additionally, the transected animals that received vehicle had a significantly lower BP throughout the monitoring period than the other three groups (*p* < 0.05). No other group comparisons were significant (*p* > 0.05). Lastly, there was also a three way interaction between time, transection, and NE, *F*_(3, 81)_ = 2.952, *p* = 0.0375. No other group comparisons were significant (*p* > 0.05).

Baseline heart rate ranged from 215 ± 19.00 to 262 ± 27.9 (mean ± SE) across groups. These differences were not statistically significant, all *F*s <1.2, *p* > 0.05. Vehicle-treated transected rats (Trans Veh) displayed lower heart rate throughout testing (Figure 3C). An ANCOVA with baseline heart rate serving as a covariate revealed a main effect of transection, *F*_(1, 27)_ = 5.370, *p* = 0.0283 (*η*^2^ = 0.141). *Post hoc* comparisons of the group means yielded a significant difference between the transected vehicle group from both sham-operated groups (*p* < 0.05). No other comparisons were significant (*p* > 0.05).

Before transection, tail blood flow ranged from 2.61 ± 1.04 to 3.26 ± 1.27 (mean ± SE) and did not differ between groups, all *F*s <1.0, *p* > 0.05. After shock treatment, sham-operated rats exhibited a rise in flow over time and this effect was not increased by NE (Figure 3D). A greater rise in tail blood flow was observed in vehicle-treated transected rats (Trans Veh) and this effect was amplified by NE (Trans NE). An ANCOVA, with baseline flow serving as the covariate, revealed a main effect of transection surgery and NE treatment, and an interaction between transection and NE treatment, all *F*s > 4.655, *p* < 0.04 (all *η*^2^ > 0.043). *Post hoc* comparisons of the group means showed that the transected group given NE (Trans NE) differed from the other three. In addition, the vehicle-treated transected group (Trans Veh) differed from both of the sham-operated groups. No other group comparisons were significant (*p* > 0.05). There was also a within subjects effect of time and an interaction between time and transection treatment, both *F*s = 2.839, *p* < 0.043.

Contused rats that were not transected (Sham) and received shock showed higher peak absorbance at the wavelength associated with hemoglobin. Pretreatment with NE had no effect (Figure 3E). An ANOVA confirmed that the main effect of transection was statistically significant, *F*_(1, 28)_ = 11.268, *p* < 0.0023 (*η*^2^ = 0.272). No other term approached significance, both *F*s <2.0, *p* > 0.05.

A similar pattern was observed with the Drabkin’s assay. Sham-operated rats showed a higher peak absorbance than transected rats, and NE had no effect (Figure 3F). An ANOVA revealed a significant main effect of transection surgery, *F*_(1, 28)_ = 4.248, *p* = 0.0487 (*η*^2^ = 0.128). No other term approached significance, both *F*s <2.0, *p* > 0.05.

Western blot confirmed the spectrophotometry analyses, demonstrating that shocked contused rats that had not received a transection (Sham) had higher concentrations of hemoglobin at the site of injury relative to the transected groups (Figure 3G). An ANOVA yielded a main effect of transection, *F*_(1, 28)_ = 37.357, *p* < 0.001 (*η*^2^ = 0.538). Neither NE treatment, nor its interaction with transection, was statistically significant, both *F*s <2.27, *p* > 0.05. *Post hoc* comparisons of group means confirmed that Transected groups differed from Sham groups (*p* < 0.05).

## Discussion

Prior work has shown that engaging pain fibers after spinal cord injury increases the area of hemorrhage at the site of injury and impairs long-term recovery (Grau et al., 2004; Turtle et al., 2019). Using noxious electrical stimulation, we have shown that disrupting communication with the brain by means of a rostral (T2) transection blocks this effect (Reynolds et al., 2019). Because electrical stimulation engages a broad range of afferent fibers, it was not clear from our earlier studies whether this brain-dependent effect involved pain fibers. The present study addressed this issue using the irritant capsaicin, which selectively engages nociceptive fibers that express the TRPV1 receptor. Capsaicin applied to one hind paw a day after injury increased hemorrhage, and this effect too was eliminated by a rostral transection (Experiment 2). This suggests that engaging this class of pain fibers is sufficient to induce hemorrhage after SCI.

The fact that engaging nociceptive fibers drives hemorrhage after SCI, and that this effect depends upon brain processes, suggests that processes that foster hemorrhage may be engaged by *perceived* pain. We have explored this possibility by testing whether pretreatment with an analgesic (morphine) blocks nociception-induced hemorrhage (Hook et al., 2007; Turtle et al., 2017). Animals received an i.p. injection of morphine at a dose (20 mg/kg) that blocked behavioral reactivity to noxious stimulation. Attenuating pain reactivity with an opiate analgesic had no effect on nociception-induced hemorrhage (Turtle et al., 2017). Likewise morphine did not block the adverse effect noxious electrical stimulation has on long-term recovery (Hook et al., 2007). These observations imply that the adverse effect of engaging nociceptive pathways is not mediated by the elicited emotional (affective) response and call into question the clinical use of opiate analgesics during the acute stage of injury. The observations suggest that an alternative state, potentially related to a sensory aspect of noxious stimulation, plays a critical role. The present study explored whether this state involved a brain-dependent surge in blood pressure and/or blood flow.

Our initial hypothesis was that afferent nociceptive stimulation may engage local processes at the site of injury that weakens the blood spinal cord barrier. An accompanying brain-dependent rise in systemic blood pressure could then fuel the infiltration of blood at the site of injury. For this purpose, we focused on systolic blood pressure, which indicates the pressure exerted against the arterial walls. Heart rate and tail blood flow were also assessed. The latter is of interest because past work suggests it can have a dual effect after injury: if the BSCB is intact, an increase in blood flow can lessen cell death by maintaining tissue perfusion (Hamamoto et al., 2007; Yuan et al., 2019); if the BSCB is compromised, and flow is excessive, it may foster blood infiltration (Strain et al., 2021).

We found that exposure to noxious intermittent electrical stimulation, but not capsaicin, produced a rise in systolic blood pressure and tail blood flow (Experiments 1 and 2). These effects were eliminated by a transection at T2. These observations have two important implications. First, there was a clear dissociation between the induction of hemorrhage and increased cardiovascular function; both noxious electrical stimulation and capsaicin drove hemorrhage, but only shock led to increased systolic BP and tail blood flow. This suggests that nociceptive input can drive hemorrhage and tissue loss independent of these processes. This conclusion is supported by a mediation analysis, which showed that pain input can drive hemorrhage in multiple ways, including a path that is independent of a rise in systolic BP or blood flow (Strain et al., 2021). Second, the nociception-induced rise in systolic BP and tail blood flow is brain-dependent and not due to lower level (T2–T6) efferent projections from the spinal cord. Interestingly, we did observe some evidence of direct efferent drive in Experiment 2, where we found capsaicin induced a rise in heart rate in both sham-operated and transected rats. Prior analyses, however, have shown that systolic BP and blood flow, not heart rate, predict the extent of nociception-induced hemorrhage after SCI (Strain et al., 2021).

Interestingly, after spinal transection, animals exhibited an increase in tail blood flow. This is consistent with other work demonstrating a disruption in autonomic regulation after a high thoracic transection (Laird et al., 2006; West et al., 2015). This may reflect, in part, a loss of tonic sympathetic activation of cutaneous arterioles involved in thermoregulation, which could increase blood flow to the tail. In addition, electrical stimulation to the tail may induce a stronger rise in blood flow, relative to capsaicin applied to a hind paw, because it has a local effect, inducing a form of neurogenic inflammation that enhances blood flow to the tail.

Elsewhere, we performed a mediational analysis that suggests an increase in general blood flow can fuel hemorrhage after SCI (Strain et al., 2021). If tail blood flow mirrors both general blood flow and spinal cord blood flow, the heightened tail flow observed after spinal transection should have been associated with amplified hemorrhage. The fact that this was not observed suggests that nociception-induced flow must interact with other processes to drive hemorrhage. Further, this second process cannot reflect a local consequence of nociceptive input, because a transection-induced rise in tail blood flow did not increase hemorrhage in animals that received noxious stimulation. The pattern of results observed here mirror those obtained when animals were pretreated with the alpha-1 adrenergic receptor inverse agonist prazosin, which blocked the pain-induced rise in systolic BP, but augmented blood flow (Strain et al., 2021). That treatment too blocked nociception-induced hemorrhage after SCI.

If brain-dependent processes drive hemorrhage after SCI because pain elicits a surge in systolic BP or general blood flow, driving this response with NE should foster hemorrhage in transected animals given shock. As observed in Experiment 1, exposure to noxious electrical stimulation increased systolic BP and hemorrhage in vehicle treated, non-transected, rats and these effects were blocked by a spinal transection. In non-transected animals, treatment with NE did not augment the shock-induced rise in systolic BP, tail blood flow, or hemorrhage (Experiment 3). These data suggests that the noxious electrical stimulation provided a form of stress that may have impacted cardiovascular function through the release of NE, resulting in a ceiling effect. In contrast, transected rats treated with NE exhibited a robust increase in systolic BP and tail blood flow, but no additional hemorrhage. The implication is that a systemic rise in systolic BP and blood flow does not substitute for the brain-dependent process(es) that drive hemorrhage after SCI; at a minimum, other processes play an essential role. Further, because all of the animals in this experiment received noxious stimulation, we can reject a simple hypothesis: that a local nociception-induced modification (e.g., the weakening of the BSCB) sets the stage for hemorrhage, which is then driven by a rise in systemic systolic BP or blood flow. Further work is needed to discover whether the missing component involves descending fibers or an additional systemic process (e.g., activation of the immune system).

We have also explored whether driving a rise in systolic BP and blood flow with NE affects the area of hemorrhage or behavioral function in non-transected contused rats (Strain et al., 2021). Interestingly, treatment with NE a day after injury adversely affected long-term recovery and increased tissue loss. The drug did not, however, induce a significant increase in the area of hemorrhage. These observations are consistent with the broader literature demonstrating that hypertension after neural injury or stroke can increase tissue loss and undermine recovery (Rawe et al., 1978; Guha et al., 1989; Jha et al., 2019). Further, it is clear that pharmacologically inducing a rise in BP soon after injury can promote hemorrhage (Soubeyrand et al., 2014; Cheung et al., 2020; Williams et al., 2020). The overall pattern of results underlines the importance of monitoring cardiovascular function after neural injury to avoid both hypo- and hypertension.

The hypothesis that motivated the present article was that brain-dependent processes drive a systemic increase in systolic blood pressure/blood flow that fuels hemorrhage at the site of injury. To explore this possibility, we employed a non-invasive procedure to assess the systemic hemodynamic response, employing a tail cuff system that emulates the assessment of blood pressure in humans with a sphygmomanometer. Importantly, the results obtained with the tail cuff system demonstrate a high correlation with the results collected using a telemetric device or catheterization (Fraser et al., 2001; Ibrahim et al., 2006; Kubota et al., 2006; Rigsby et al., 2007; Brailoiu et al., 2014; Holditch et al., 2015). While these alternative measures have advantages, it is clear that the tail-cuff system used here, and in prior work (Strain et al., 2021), can reliably resolve hemodynamic effects of nociceptive stimulation. Further, statistical modeling has shown that our measures of systolic blood pressure and tail blood flow have a significant relation to the extent of hemorrhage and acute drop in locomotor function (Strain et al., 2021). While these observations suggest that we have a good index of the systemic response to nociceptive stimulation, we must acknowledge that SCI brings a local increase in intraspinal pressure that decreases perfusion pressure, reducing local blood flow and tissue perfusion. Recent evidence suggests that this local rise in intraspinal pressure can fuel hemorrhage after injury, which can be attenuated by increasing perfusion pressure (Williams et al., 2020).

A limitation of the current article stems from our inability to directly measure spinal cord blood flow. Using the tail cuff system, we found interesting correlations between tail blood flow and spinal hemorrhage, suggesting a potential parallel in blood flow between the two tissues. Under normal conditions, parallels between changes in tail blood flow (which is regulated by sympathetic activity and inflammation, including neurogenic inflammation) and spinal cord blood flow (which is autoregulated) would not be expected and might even have an inverse relationship. However, the possibility that, after SCI, increased tail blood flow parallels increased spinal cord blood flow, which then fuels spinal hemorrhage after noxious stimulation, is consistent with two observations. First, SCI can impair autoregulation in spinal vasculature (Ohashi et al., 1996). Second, spinal contusion at T10-T11 may decrease excitation of preganglionic neurons that drive sympathetic activity in the tail. These changes might allow noxious stimulation of the tail or a hind paw to produce vasodilation and increased blood flow both in the stimulated peripheral tissue (classic neurogenic inflammation) and possibly across many segments of the spinal cord. Further work will need to directly investigate whether and how noxious stimulation after SCI enhances spinal cord blood flow.

It is possible that a spinal cord transection has a beneficial effect, in part, because it provides a kind of durotomy that relieves the local intraspinal pressure and thereby enhances blood flow to the injured tissue (Streijger et al., 2021). While this is an important consideration, the beneficial effect of a durotomy presumably declines with distance from the injury and, for this reason, it is not clear whether a remote cut (approximately 4 cm rostral to the site of injury) would have a significant effect at the site of injury. Further work will be needed to explore this possibility.

Importantly, our conclusion that rostral processes play a role is based on converging operations. First, a pharmacological transection, induced by slowing infusing the anesthetic lidocaine at T2, blocks nociception-induced hemorrhage (Davis et al., 2020). Importantly, lidocaine treatment also attenuated the adverse effect noxious stimulation has on long-term recovery. The observations are clinically important because they show that a pharmacological blockade can have a protective effect; the findings are methodologically important because they show a similar pattern of results is obtained in the absence of a second surgery (required to perform a rostral transection). Other recent studies have shown that placing animals in a state akin to a medically-induced coma blocks both nociception-induced hemorrhage and the rise in systolic blood pressure and tail blood flow (Davis et al., 2017). Taken together, the results suggest blocking the activation of a brain-dependent process by incoming nociceptive signals has a protective effect that reduces the extent of hemorrhage/secondary injury.

It is clear from these findings that nociceptive fibers activated by tail shock can engage brain processes that fuel hemorrhage at the site of injury. Paradoxically, this implies that a less severe injury (that spares ascending fibers) may lead to additional tissue loss at the site of injury (due to the destructive effect of brain-dependent processes). Supporting this, we have shown that the adverse effect of pain input after SCI varies inversely with injury severity, being greater after a light to moderate (6.25–12.5 mm weight drop) injury (Reynolds et al., 2019). Taken together, our results show that spared ascending fibers can engage rostral processes that can fuel tissue loss after SCI. What remains unclear is how these processes drive hemorrhage. Does this reflect a systemic process, the effect of descending fibers, or an interaction between the two?

In summary, the results provide further evidence that nociceptive stimulation after SCI can fuel hemorrhage and that this effect depends upon processes rostral to T2. Nociceptive stimulation can also engage a brain-dependent rise in systolic BP and tail blood flow, with the magnitude of this effect depending upon stimulus type. Aversive intermittent electrical stimulation engaged a robust cardiovascular response that remained elevated for 3 h. In contrast, selectively activating nociceptive fibers that the express TRPV1 receptor with capsaicin had a negligible effect on systolic BP and tail blood flow yet drove robust hemorrhage. Importantly, driving a rise in systolic BP or tail blood flow with NE was not associated with an increase in the extent of hemorrhage after SCI, suggesting that other processes play a critical role. Further work is needed to identify these mechanisms.

## Data Availability Statement

The raw data supporting the conclusions of this article will be made available by the authors, without undue reservation.

## Ethics Statement

The animal study was reviewed and approved by University Laboratory Animal Care Committee at Texas A&M University.

## Author Contributions

The study was conducted by GF, MS, Y-JH, JR, JD, and MH. The data were analyzed by GF and JG. The article was written by GF and JG with input from CW. All authors contributed to the article and approved the submitted version.

## Conflict of Interest

Y-JH is employed by Sundia MediTech Co., Ltd. The remaining authors declare that the research was conducted in the absence of any commercial or financial relationships that could be construed as a potential conflict of interest.

## Publisher’s Note

All claims expressed in this article are solely those of the authors and do not necessarily represent those of their affiliated organizations, or those of the publisher, the editors and the reviewers. Any product that may be evaluated in this article, or claim that may be made by its manufacturer, is not guaranteed or endorsed by the publisher.

## References

[B1] Alizadeh A. Dyck S. M. Karimi-Abdolrezaee S. (2019). Traumatic spinal cord injury: an overview of pathophysiology, models and acute injury mechanisms. Front. Neurol. 10:282. 10.3389/fneur.2019.00282 30967837 PMC6439316

[B2] Basso D. M. Beattie M. S. Bresnahan J. C. (1995). A sensitive and reliable locomotor rating scale for open field testing in rats. J. Neurotrauma 12, 1–21. 10.1089/neu.1995.12.1 7783230

[B3] Baumbauer K. M. Hoy K. C. Huie J. R. Hughes A. J. Woller S. A. Puga D. A.. (2008). Timing in the absence of supraspinal input I: Variable, but not fixed, spaced stimulation of the sciatic nerve undermines spinally-mediated instrumental learning. Neuroscience 155, 1030–1047. 10.1016/j.neuroscience.2008.07.003 18674601 PMC2633135

[B4] Beattie M. S. Hermann G. E. Rogers R. C. Bresnahan J. C. (2002). “Cell death in models of spinal cord injury,” in Spinal Cord Trauma: Regeneration, Neural Repair and Functional Recovery, eds McKerracher L. Doucet G. Rossignol S. (Amsterdam: Elsevier Science BV), 37–47.

[B5] Brailoiu G. C. Deliu E. Rabinowitz J. E. Tilley D. G. Koch W. J. Brailoiu E. (2014). Urotensin II promotes vagal-mediated bradycardia by activating cardiac-projecting parasympathetic neurons of nucleus ambiguus. J. Neurochem. 129, 628–636. 10.1111/jnc.12679 24521102 PMC4000260

[B6] Canon S. Shera A. Phan N. M. H. Lapicz L. Scheidweiler T. Batchelor L.. (2015). Autonomic dysreflexia during urodynamics in children and adolescents with spinal cord injury or severe neurologic disease. J. Pediatr. Urol. 11, P32.E1–32.E4. 10.1016/j.jpurol.2014.08.011 25697979

[B7] Cheung A. Streijger F. So K. Okon E. B. Manouchehri N. Shortt K.. (2020). The relationship between early vasopressor administration and spinal cord hemorrhage in a porcine model of acute traumatic spinal cord injury. J. Neurotrauma 37, 1696–1707. 10.1089/neu.2019.6781 32233727

[B8] Choudhri T. F. Hoh B. L. Solomon R. A. Connolly E. S. Pinsky D. J. (1997). Use of a spectrophotometric hemoglobin assay to objectively quantify intracerebral hemorrhage in mice. Stroke 28, 2296–2302. 10.1161/01.str.28.11.2296 9368579

[B9] Crown E. D. Ferguson A. R. Joynes R. L. Grau J. W. (2002). Instrumental learning within the spinal cord: IV. Induction and retention of the behavioral deficit observed after noncontingent shock. Behav. Neurosci. 116, 1032–1051. 10.1037//0735-7044.116.6.1032 12492302

[B10] Crown E. D. Grau J. W. (2005). Evidence that descending serotonergic systems protect spinal cord plasticity against the disruptive effect of uncontrollable stimulation. Exp. Neurol. 196, 164–176. 10.1016/j.expneurol.2005.07.016 16139268

[B12] Davis J. A. Bopp A. C. Henwood M. K. Baine R. E. Cox C. C. Grau J. W. (2020). Pharmacological transection of brain-spinal cord communication blocks pain-induced hemorrhage and locomotor deficits after spinal cord injury in rats. J. Neurotrauma 37, 1729–1739. 10.1089/neu.2019.6973 32368946 PMC7368389

[B11] Davis J. Haung Y. J. Strain M. Reynolds J. Grau J. (2017). Pain-induced hemorrhage after spinal cord injury is blocked by pentobarbital anesthesia. J. Neurotrauma 34:A121. 10.1089/neu.2017.29011

[B13] Ducker T. B. Kindt G. W. Kempe L. G. (1971). Pathological findings in acute experimental spinal cord trauma. J. Neurosurg. 35, 700–708. 10.3171/jns.1971.35.6.0700 5000662

[B14] Eldahan K. C. Rabchevsky A. G. (2018). Autonomic dysreflexia after spinal cord injury: Systemic pathophysiology and methods of management. Auton. Neurosci. 209, 59–70. 10.1016/j.autneu.2017.05.002 28506502 PMC5677594

[B15] Feng M. Whitesall S. Zhang Y. Beibel M. D’Alecy L. DiPetrillo K. (2008). Validation of volume-pressure recording tail-cuff blood pressure measurements. Am. J. Hypertens. 21, 1288–1291. 10.1038/ajh.2008.301 18846043

[B16] Ferguson A. R. Crown E. D. Grau J. W. (2006). Nociceptive plasticity inhibits adaptive learning in the spinal cord. Neuroscience 141, 421–431. 10.1016/j.neuroscience.2006.03.029 16678969

[B17] Fraser T. B. Turner S. W. Mangos G. J. Ludbrook J. Whitworth J. A. (2001). Comparison of telemetric and tail-cuff blood pressure monitoring in adrenocorticotrophic hormone-treated rats. Clin. Exp. Pharmacol. Physiol. 28, 831–835. 10.1046/j.1440-1681.2001.03531.x 11553024

[B18] Grau J. W. Huie J. R. Garraway S. M. Hook M. A. Crown E. D. Baumbauer K. M.. (2012). Impact of behavioral control on the processing of nociceptive stimulation. Front. Physiol. 3:262. 10.3389/fphys.2012.00262 22934018 PMC3429038

[B19] Grau J. W. Washburn S. N. Hook M. A. Ferguson A. R. Crown E. D. Garcia G.. (2004). Uncontrollable stimulation undermines recovery after spinal cord injury. J. Neurotrauma 21, 1795–1817. 10.1089/neu.2004.21.1795 15684770

[B20] Gruner J. A. (1992). A monitored contusion model of spinal-cord injury in the rat. J. Neurotrauma 9, 123–126. 10.1089/neu.1992.9.123 1404425

[B21] Guha A. Tator C. H. Rochon J. (1989). Spinal cord blood flow and systemic blood pressure after experimental spinal cord injury in rats. Stroke 20, 372–377. 10.1161/01.str.20.3.372 2922776

[B22] Hamamoto Y. Ogata T. Morino T. Hino M. Yamamoto H. (2007). Real-time direct measurement of spinal cord blood flow at the site of compression: relationship between blood flow recovery and motor deficiency in spinal cord injury. Spine (Phila Pa 1976) 32, 1955–1962. 10.1097/BRS.0b013e3181316310 17700440

[B23] Hardebo J. E. Beley A. (1984). Influence of blood-pressure on blood-brain barrier function in brain ischemia. Acta Neurol. Scand. 70, 356–359. 10.1111/j.1600-0404.1984.tb00836.x 6507045

[B24] Hathway G. J. Vega-Avelaira D. Moss A. Ingram R. Fitzgerald M. (2009). Brief, low frequency stimulation of rat peripheral C-fibres evokes prolonged microglial-induced central sensitization in adults but not in neonates. Pain 144, 110–118. 10.1016/j.pain.2009.03.022 19410369 PMC2702711

[B25] Hausmann O. N. (2003). Post-traumatic inflammation following spinal cord injury. Spinal Cord 41, 369–378. 10.1038/sj.sc.3101483 12815368

[B26] Heistad D. D. Marcus M. L. (1979). Effect of sympathetic stimulation on permeability of the blood-brain barrier to albumin during acute hypertension in cats. Circ. Res. 45, 331–338. 10.1161/01.res.45.3.331 455598

[B27] Holditch S. J. Schreiber C. A. Nini R. Tonne J. M. Peng K. W. Geurts A.. (2015). B-type natriuretic peptide deletion leads to progressive hypertension, associated organ damage and reduced survival: novel model for human hypertension. Hypertension 66, 199–210. 10.1161/HYPERTENSIONAHA.115.05610 26063669 PMC4467451

[B28] Hook M. A. Huie J. R. Grau J. W. (2008). Peripheral inflammation undermines the plasticity of the isolated spinal cord. Behav. Neurosci. 122, 233–249. 10.1037/0735-7044.122.1.233 18298266 PMC2665167

[B29] Hook M. A. Liu G. T. Washburn S. N. Ferguson A. R. Bopp A. C. Huie J. R.. (2007). The impact of morphine after a spinal cord injury. Behav. Brain Res. 179, 281–293. 10.1016/j.bbr.2007.02.035 17383022 PMC1965266

[B30] Hook M. A. Woller S. A. Bancroft E. Aceves M. Funk M. K. Hartman J.. (2017). Neurobiological effects of morphine after spinal cord injury. J. Neurotrauma 34, 632–644. 10.1089/neu.2016.4507 27762659 PMC5286553

[B31] Huang Y. J. Lee K. H. Murphy L. Garraway S. M. Grau J. W. (2016). Acute spinal cord injury (SCI) transforms how GABA affects nociceptive sensitization. Exp. Neurol. 285, 82–95. 10.1016/j.expneurol.2016.09.005 27639636 PMC5926208

[B32] Ibrahim J. Berk B. C. Hughes A. D. (2006). Comparison of simultaneous measurements of blood pressure by tail-cuff and carotid arterial methods in conscious spontaneously hypertensive and Wistar-Kyoto rats. Clin. Exp. Hypertens. 28, 57–72. 10.1080/10641960500386817 16443565

[B33] Ito U. Ohno K. Yamaguchi T. Takei H. Tomita H. Inaba Y. (1980). Effect of hypertension on blood-brain barrier change after restoration of blood flow in post-ischemic gerbil brains. An elecromicroscopic study. Stroke 11, 606–611. 10.1161/01.str.11.6.606 7210066

[B34] Jha R. M. Desai S. M. Zusman B. E. Koleck T. A. Puccio A. M. Okonkwo D. O.. (2019). Downstream TRPM4 polymorphisms are associated with intracranial hypertension and statistically interact with ABCC8 polymorphisms in a prospective cohort of severe traumatic brain injury. J. Neurotrauma 36, 1804–1817. 10.1089/neu.2018.6124 30484364 PMC6551973

[B35] Johnston D. T. Lout E. Baine R. E. Grau J. W. (2021). National neurotrauma symposium, virtual, 2021. J. Neurotrauma 38, A.1–A.132. 10.1089/neu.2021.29111

[B36] Karlsson A. K. (1999). Autonomic dysreflexia. Spinal Cord 37, 383–391. 10.1038/sj.sc.3100867 10432257

[B37] Krassioukov A. V. Furlan J. C. Fehlings M. G. (2003). Autonomic dysreflexia in acute spinal cord injury: An under-recognized clinical entity. J. Neurotrauma 20, 707–716. 10.1089/089771503767869944 12965050

[B38] Kubota Y. Umegaki K. Kagota S. Tanaka N. Nakamura K. Kunitomo M.. (2006). Evaluation of blood pressure measured by tail-cuff methods (without heating) in spontaneously hypertensive rats. Biol. Pharm. Bull. 29, 1756–1758. 10.1248/bpb.29.1756 16880638

[B39] Laird A. S. Carrive P. Waite P. M. E. (2006). Cardiovascular and temperature changes in spinal cord injured rats at rest and during autonomic dysreflexia. J. Physiol. 577, 539–548. 10.1113/jphysiol.2006.116301 16973703 PMC1890430

[B40] Lamotte R. H. Shain C. N. Simone D. A. Tsai E. F. P. (1991). Neurogenic hyperalgesia: psychophysical studies of underlying mechanisms. J. Neurophysiol. 66, 190–211. 10.1152/jn.1991.66.1.190 1919666

[B41] Lee J. Y. Choi H. Y. Na W. H. Ju B. G. Yune T. Y. (2014). Ghrelin inhibits BSCB disruption/hemorrhage by attenuating MMP-9. and SUR1/TrpM4 expression and activation after spinal cord injury. Biochim. Biophys. Acta 1842, 2403–2412. 10.1016/j.bbadis.2014.09.006 25261791

[B42] Lee J. Y. Choi H. Y. Na W. H. Ju B. G. Yune T. Y. (2015). 17 beta-estradiol inhibits MMP-9. and SUR1/TrpM4 expression and activation and thereby attenuates BSCB disruption/hemorrhage after spinal cord injury in male rats. Endocrinology 156, 1838–1850. 10.1210/en.2014-1832 25763638

[B43] Lee J. Y. Choi H. Y. Park C. S. Ju B. G. Yune T. Y. (2018). Mithramycin a improves functional recovery by inhibiting bscb disruption and hemorrhage after spinal cord injury. J. Neurotrauma 35, 508–520. 10.1089/neu.2017.5235 29048243

[B44] Lindan R. Joiner E. Freehafer A. A. Hazel C. (1980). Incidence and clinical features of autonomic dysreflexia in patients with spinal cord injury. Paraplegia 18, 285–292. 10.1038/sc.1980.51 7443280

[B45] Lucin K. M. Sanders V. M. Jones T. B. Malarkey W. B. Popovich P. G. (2007). Impaired antibody synthesis after spinal cord injury is level dependent and is due to sympathetic nervous system dysregulation. Exp. Neurol. 207, 75–84. 10.1016/j.expneurol.2007.05.019 17597612 PMC2023967

[B46] Lucin K. M. Sanders V. M. Popovich P. G. (2009). Stress hormones collaborate to induce lymphocyte apoptosis after high level spinal cord injury. J. Neurochem. 110, 1409–1421. 10.1111/j.1471-4159.2009.06232.x 19545280 PMC2737096

[B47] Marsh D. R. Weaver L. C. (2004). Autonomic dysreflexia, induced by noxious or innocuous stimulation, does not depend on changes in dorsal horn substance, p. J. Neurotrauma 21, 817–828. 10.1089/0897715041269605 15253807

[B48] Mautes A. E. M. Weinzierl M. R. Donovan F. Noble L. J. (2000). Vascular events after spinal cord injury: contribution to secondary pathogenesis. Phys. Ther. 80, 673–687. 10.1093/ptj/80.7.673 10869130

[B49] McVeigh J. F. (1923). Experimental cord crushes with especial reference to the mechanical factors involved and subsequent changes in the areas of the cord affected. Arch. Surg. 7, 573–600. 10.1001/archsurg.1923.01120030106004

[B50] Nielson J. L. Paquette J. Liu A. W. Guandique C. F. Tovar C. A. Inoue T.. (2015). Topological data analysis for discovery in preclinical spinal cord injury and traumatic brain injury. Nat. Commun. 6:8581. 10.1038/ncomms9581 26466022 PMC4634208

[B51] Ohashi T. Morimoto T. Kawata K. Yamada T. Sakaki T. (1996). Correlation between spinal cord blood flow and arterial diameter following acute spinal cord injury in rats. Acta Neurochir. (Wien) 138, 322–329. 10.1007/BF01411744 8861702

[B53] Rabchevsky A. G. (2006). Segmental organization of spinal reflexes mediating autonomic dysreflexia after spinal cord injury. Prog. Brain Res. 152, 265–274. 10.1016/S0079-6123(05)52017-X 16198706 PMC3529572

[B52] Rabchevsky A. G. Patel S. P. Lyttle T. S. Eldahan K. C. O’Dell C. R. Zhang Y.. (2012). Effects of gabapentin on muscle spasticity and both induced as well as spontaneous autonomic dysreflexia after complete spinal cord injury. Front. Physiol. 3:329. 10.3389/fphys.2012.00329 22934077 PMC3429097

[B54] Rawe S. E. Lee W. A. Perot P. L. Jr. (1978). The histopathology of experimental spinal cord trauma. The effect of systemic blood pressure. J. Neurosurg. 48, 1002–1007. 10.3171/jns.1978.48.6.1002 660233

[B55] Reynolds J. A. Henwood M. K. Turtle J. D. Baine R. E. Johnston D. T. Grau J. W. (2019). Brain-dependent processes fuel pain-induced hemorrhage after spinal cord injury. Front. Syst. Neurosci. 13:44. 10.3389/fnsys.2019.00044 31551720 PMC6746957

[B56] Rigsby C. S. Burch A. E. Ogbi S. Pollock D. M. Dorrance A. M. (2007). Intact female stroke-prone hypertensive rats lack responsiveness to mineralocorticoid receptor antagonists. Am. J. Physiol. Regul. Integr. Comp. Physiol. 293, R1754–R1763. 10.1152/ajpregu.00145.2007 17670862 PMC2804103

[B57] Sadie W. C. (1920). A method for the determination of methemoglobin in blood. J. Biol. Chem. 41, 237–241.

[B58] Simard J. M. Woo S. K. Aarabi B. Gerzanich V. (2013). The Sur1-Trpm4 channel in spinal cord injury. J. Spine S4:002. 10.4172/2165-7939.S4-002 24834370 PMC4019017

[B59] Simard J. M. Woo S. K. Norenberg M. D. Tosun C. Chen Z. Ivanova S.. (2010). Brief suppression of abcc8 prevents autodestruction of spinal cord after trauma. Sci. Transl. Med. 2:28ra29. 10.1126/scitranslmed.3000522 20410530 PMC2903041

[B60] Simone D. A. Baumann T. K. LaMotte R. H. (1989). Dose-dependent pain and mechanical hyperalgesia in humans after intradermal injection of capsaicin. Pain 38, 99–107. 10.1016/0304-3959(89)90079-1 2780068

[B61] Snow J. C. Sideropoulos H. D. Kripke B. J. Freed M. M. Shah N. K. Schlesinger R. M. (1978). Autonomic hyperreflexia during cystoscopy in patients with high spinal cord injuries. Paraplegia 15, 327–332. 10.1038/sc.1977.49 625432

[B62] Soubeyrand M. Dubory A. Laemmel E. Court C. Vicaut E. Duranteau J. (2014). Effect of norepinephrine on spinal cord blood flow and parenchymal hemorrhage size in acute-phase experimental spinal cord injury. Eur. Spine J. 23, 658–665. 10.1007/s00586-013-3086-9 24232597 PMC3940804

[B63] Strain M. M. Johnston D. T. Baine R. E. Reynolds J. Huang Y. J. Henwood M. K.. (2021). Hemorrhage and locomotor deficits induced by pain input after spinal cord injury are partially mediated by changes in hemodynamics. J. Neurotrauma. [Online ahead of print]. 10.1089/neu.2021.0219 34652956 PMC8713547

[B64] Streijger F. Kim K. T. So K. Manouchehri N. Shortt K. Okon E. B.. (2021). Duraplasty in traumatic thoracic spinal cord injury: impact on spinal cord hemodynamics, tissue metabolism, histology and behavioral recovery using a porcine model. J. Neurotrauma 38, 2937–2955. 10.1089/neu.2021.0084 34011164

[B65] Thompson S. W. King A. E. Woolf C. J. (1990). Activity-dependent changes in rat ventral horn neurons *in vitro*; summation of prolonged afferent evoked postsynaptic depolarizations produce a d-2.-Amino-5.-Phosphonovaleric acid sensitive windup. Eur. J. Neurosci. 2, 638–649. 10.1111/j.1460-9568.1990.tb00453.x 12106298

[B66] Turtle J. D. Henwood M. K. Strain M. M. Huang Y. J. Miranda R. C. Grau J. W. (2019). Engaging pain fibers after a spinal cord injury fosters hemorrhage and expands the area of secondary injury. Exp. Neurol. 311, 115–124. 10.1016/j.expneurol.2018.09.018 30268767 PMC6530785

[B67] Turtle J. D. Strain M. M. Aceves M. Huang Y. J. Reynolds J. A. Hook M. A.. (2017). Pain input impairs recovery after spinal cord injury: treatment with lidocaine. J. Neurotrauma 34, 1200–1208. 10.1089/neu.2016.4778 27912032 PMC5359686

[B68] Turtle J. D. Strain M. M. Reynolds J. A. Huang Y. J. Lee K. H. Henwood M. K.. (2018). Pain input after spinal cord injury (SCI) undermines long-term recovery and engages signal pathways that promote cell death. Front. Syst. Neurosci. 12:27. 10.3389/fnsys.2018.00027 29977195 PMC6021528

[B69] van kampen E. Zijlstra W. G. (1961). Standardization of hemoglobin II. The hemiglobincyanide method. Clin. Chim. Acta 6, 538–544. 10.1016/0009-8981(61)90145-0 14453500

[B70] West C. R. Popok D. Crawford M. A. Krassioukov A. V. (2015). Characterizing the temporal development of cardiovascular dysfunction in response to spinal cord injury. J. Neurotrauma 32, 922–930. 10.1089/neu.2014.3722 25630034

[B71] West C. R. Squair J. W. McCracken L. Currie K. D. Somvanshi R. Yuen V.. (2016). Cardiac consequences of autonomic dysreflexia in spinal cord injury. Hypertension 68, 1281–1289. 10.1161/HYPERTENSIONAHA.116.07919 27698067

[B72] Williams A. M. Manouchehri N. Erskine E. Tauh K. So K. Shortt K.. (2020). Cardio-centric hemodynamic management improves spinal cord oxygenation and mitigates hemorrhage in acute spinal cord injury. Nat. Commun. 11:5209. 10.1038/s41467-020-18905-8 33060602 PMC7562705

[B73] Yuan X. C. Wu Q. B. Wang P. Jing Y. L. Yao H. J. Tang Y.. (2019). Exosomes derived from pericytes improve microcirculation and protect blood-spinal cord barrier after spinal cord injury in mice. Front. Neurosci. 13:319. 10.3389/fnins.2019.00319 31040762 PMC6476953

